# The clinical prognostic value of lncRNA FAM83H-AS1 in cancer patients: a meta-analysis

**DOI:** 10.1186/s12935-020-1148-8

**Published:** 2020-03-06

**Authors:** Qin Yang, Jie Wang, Pingyong Zhong, Tinggang Mou, Hao Hua, Pan Liu, Fei Xie

**Affiliations:** 1 Department of Gastroenterology, The First People’s Hospital of Neijiang, Neijiang, Sichuan China; 2 Department of Hepatic-Biliary-Pancreatic Surgery, The First People’s Hospital of Neijiang, No. 31, Tuozhong Lane, Jiaotong Road, Neijiang, 641000 Sichuan China

**Keywords:** Cancer, Overall survival, Prognosis, Long non-coding RNA, FAM83H-AS1

## Abstract

**Background:**

Family with sequence similarity 83 member H antisense RNA 1 (FAM83H-AS1) is a novel long non-coding RNA. Increasing studies have reported that FAM83H-AS1 is abnormally expressed in a variety of tumors and is associated with poor outcome. However, the clinical prognostic significance of lncRNA FAM83H-AS1 in tumors is not completely known.

**Methods:**

In this meta-analysis, literature was collected up until February 5, 2020 through multifarious retrieval strategies by searching through electronic databases of PubMed, Cochrane Library, EMBASE, Medline, Web of Science, CNKI, Weipu, and Wanfang. A total of 14 studies that met the inclusion criteria with relevant clinical data and prognostic information were included in the meta-analysis.

**Results:**

The combined results revealed that high expression of FAM83H-AS1 was associated with poor overall survival (OS) (HR = 1.63, 95% CI 1.24–2.14, *P *= 0.0004) in a variety of cancers. Additionally, upregulated FAM83H-AS1 expression was significantly correlated with tumor TNM stage (III/IV vs. I/II, OR = 2.40, 95% CI 1.36–4.23, *P *= 0.003) and lymph node metastasis (positive vs. negative, OR = 1.70, 95% CI 1.14–2.52, *P *= 0.008) in patients with cancer.

**Conclusions:**

Our results of this meta-analysis indicated that elevated FAM83H-AS1 expression could predict poor prognosis in patients with cancer and suggested that FAM83H-AS1 might serve as a novel biomarker for cancer.

## Introduction

Long non-coding RNA (lncRNA) is a type of non-coding regulatory RNA that is more than 200 nucleotides in length without protein-coding ability [1]. LncRNAs have been a focus of intense investigation in life science research in the past decade. Increasing studies have shown that lncRNAs play an important role in various activities such as epigenetic regulation, cell cycle regulation, cell differentiation regulation and dosage compensation [2]. Dysregulated lncRNA expression is closely related to multiple human diseases, including a variety of cancers, suggesting a potential function of these lncRNAs as biomarkers for diagnosis, prognosis, cancer stage, and response to therapy [3–5].

Several studies have indicated that abnormal expression of the lncRNA family with sequence similarity 83 member H antisense RNA 1(FAM83H-AS1) is associated with poor prognosis in patients with cancer. Ma et al. revealed that increased FAM83H-AS1 expression was associated with shorter patient overall survival (OS) in patients with hepatocellular carcinoma [6]. In gastric cancer, upregulated FAM83H-AS1 was a risk factor related to OS and disease-free survival, and FAM83H-AS1 might function as an oncogene [7]. Another study showed that patients with ovarian cancer were more easily accompanied by high expression of FAM83H-AS1, which was correlated with tumor pathological grade, tumor lymph node metastasis (TNM) stage and distant metastasis [8]. Overexpressed FAM83H-AS1 exhibited oncogenic functions in glioma by suppressing expression of CDKN1A, which encoded a key factor that regulated the G1 phase of the cell cycle, leading to increased cell proliferation [9]. Furthermore, high expression of FAM83H-AS1 was related to advanced clinical stage in bladder cancer and FAM83H-AS1 was more likely to lead to invasion of muscle layer, suggesting FAM83H-AS1 could be an independent poor prognostic factor for OS in bladder cancer patients [10]. However, no systematic meta-analysis has so far clarified the prognostic value of FAM83H-AS1 in these cancers.

Therefore, we performed a meta-analysis to explore the clinical prognostic value of the lncRNA FAM83H-AS1 in patients with cancers.

## Methods

### Literature search

A comprehensive and systematic literature search was performed up until February 5, 2020 through multifarious retrieval strategies. Two authors (Qin Yang and Jie Wang) were responsible for completing the search in electronic databases including PubMed, Cochrane Library, EMBASE, Medline, Web of Science, CNKI, Weipu, and Wanfang. The following keywords were used for searching, including: “(((((family with sequence similarity 83 member H-antisense RNA 1) OR family with sequence similarity 83 member H-AS1)) OR ((((FAM83H-AS1) OR long non-coding RNA FAM83H-AS1) OR FAM83H antisense RNA 1) OR onco-lncRNA-3))) AND ((((((((cancer) OR tumor) OR carcinoma) OR neoplasm)) AND ((((prognosis) OR survival) OR diagnosis) OR clinicopathological)))).”

### Inclusion and exclusion criteria

The criteria for inclusion of literature in the study were as follows: (1) studies with a definite diagnosis or histopathological diagnosis of cancer patients; (2) studies examining prognostic characteristics of lncRNA FAM83H-AS1 in patients with malignant tumor; and (3) studies with sufficient information to calculate the combined hazard risk (HR) and 95% confidence interval (CI).

The criteria for exclusion were as follows: (1) studies without prognostic outcomes; (2) duplicate publications; and (3) non-human studies, letters, case reports, letters, review articles and other studies without survival data.

### Data extraction and quality assessment

Three authors (Hao Hua, Pingyong Zhong, Tinggang Mou) independently completed data extraction and reached a consensus. After a set of literature was established according to the above criteria, the following information was successively extracted: author, country, year of publication, tumor type, tumor size, follow-up time, detection method and cut-off value. The number of patients in each group was divided according to the presence or absence of lymph node metastasis, distant metastasis, tumor size, TNM stage, and the number of patients with high or low FAM83H-AS1 expression in each group.

If only Kaplan–Meier curves were available, we extracted the data from the graph survival chart using Engauge Digitizer V4.1 to estimate the survival time and HR. If a study reported data from multivariate or univariate analyses of OS, the former one was applied directly. The Newcastle–Ottawa Quality Assessment Scale (NOS) was performed to evaluate the quality of each eligible study with a score ranging from 0 to 9 points [11].

### Statistical analysis

Review Manager (RevMan) 5.3 software was used to combine HR or odds ratio for this meta-analysis. Stata 14 software was used to estimate the publication bias of this meta-analysis. The heterogeneity of results was estimated by *Q* test and *I*^2^ statistics. The fixed-effects model was selected for data analysis when *I*^2^< 50%. The random-effects model was used for data analysis when the heterogeneity was obvious (*I*^2^> 50%) [12]. If the result indicated HR > 1, lncRNA overexpression was significantly correlated with survival difference. HR < 1 indicated that lncRNA overexpression predicted long survival.

## Results

### Characteristics and basic information in eligible literature

**Fig. 1:**
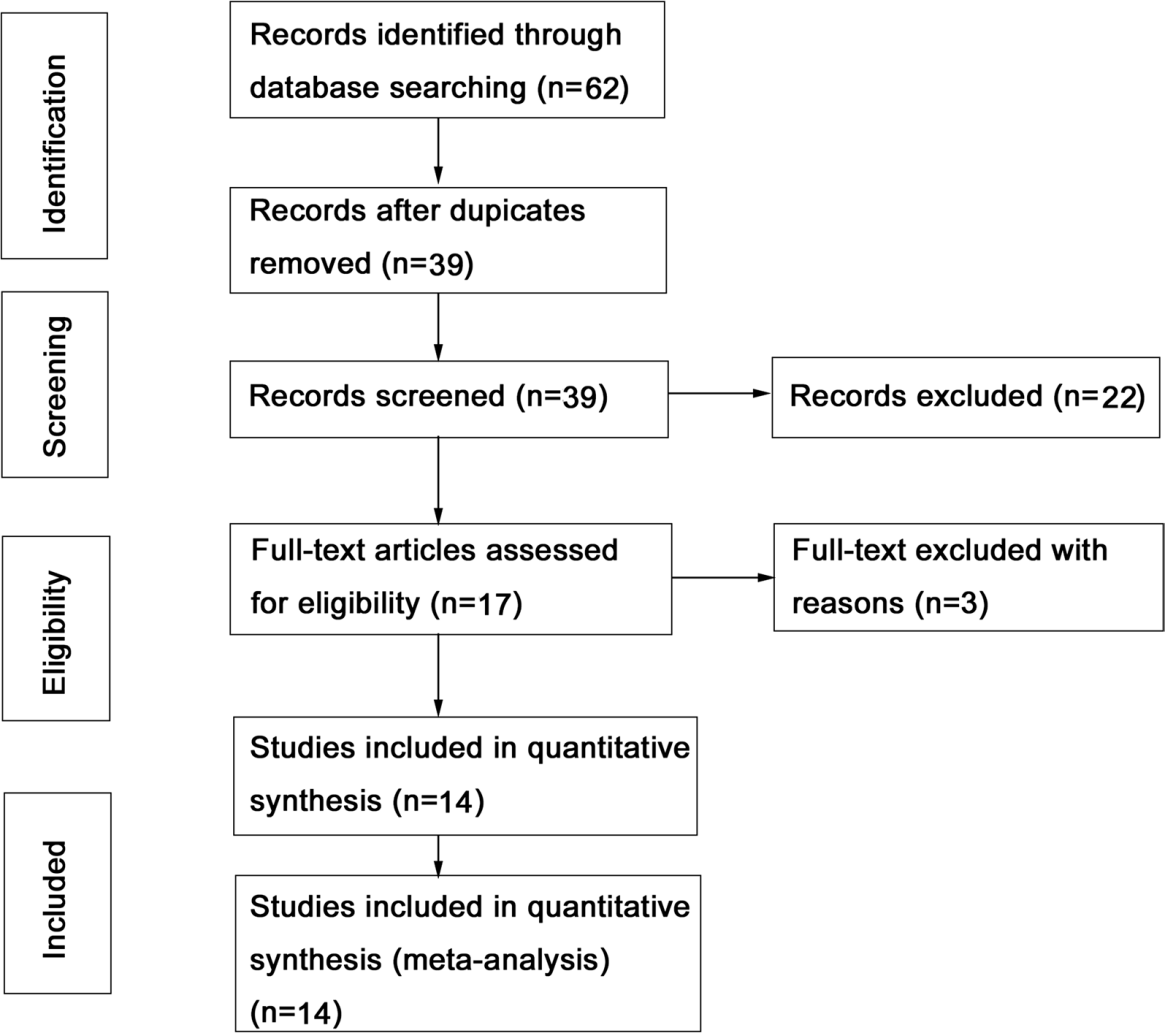
Flow diagram of the literatures selection procedure in this meta-analysis

By searching the electronic databases from PubMed, Cochrane Library, EMBASE, Medline, Web of Science, CNKI, Weipu, and Wanfang, we preliminarily retrieved 62 relevant studies. We carefully reviewed the title, abstract, and full content and evaluated whether the study contained available clinical data. A total of 14 studies published between 2016 and 2020 were eligible to be included in this meta-analysis (Fig. 1). The 14 studies contained 2818 patients from three countries including China, United States and Iran. We mainly analyzed nine different types of tumors, including pancreatic ductal adenocarcinoma [13], gastric cancer [7, 14, 15], ovarian cancer [8, 16], glioma [9], colorectal carcinoma [17, 18], bladder cancer [10], hepatocellular carcinoma [6], breast cancer [19], and lung cancer [20]. The expression of FAM83H-AS1 was detected by quantitative real-time fluorescent PCR in all studies. To distinguish the expression levels of FAM83H-AS1, the cut-off value was used. All studies selected median values and used OS to estimate patient survival. Ten studies contained detailed clinical prognosis information to analyze clinical outcome. Detailed information was shown in Table 1.

**Table 1:** The main characteristics of the eligible literatures included in the meta-analysis

Study	Region	Tumor type	Sample size	TNM stage	FAM83H-AS1 expression		Cut-off value	Detection method	Outcome measure	NOS
					High	Low				
Arnes et al. [13]	United States	PDAC	49	NA	25	24	Median	qRT-PCR	OS	5
Baratieh et al. [14]	Iran	GC	74	I–IV	37	37	Median	qRT-PCR	OS	7
		BRCA	382	NA	191	191	Median	qRT-PCR	OS	5
		KIRP	72	NA	36	36	Median	qRT-PCR	OS	5
		OC	184	NA	92	92	Median	qRT-PCR	OS	5
Bi et al. [9]	China	Glioma	187	I–IV	29	19	Median	qRT-PCR	OS	7
Da et al. [7]	China	GC	107	I–IV	53	54	Median	qRT-PCR	OS, DFS	7
Dou et al. [16]	China	OC	80	I–IV	38	42	Median	qRT-PCR	OS	7
Gong et al. [8]	China	OC	100	I–IV	48	52	Median	qRT-PCR	OS	7
Lu et al. [18]	China	CRC	62	I–IV	31	31	Median	qRT-PCR	OS	7
Ma et al. [21]	China	HCC	66	I–IV	32	34	Median	qRT-PCR	OS	7
Shan et al. [10]	China	BLCA	96	I–IV	48	48	Median	qRT-PCR	OS	7
Wang et al. [15]	China	GC	168	NA	84	84	Median	qRT-PCR	NA	6
Yang et al. [32]	China	CRC	87	I–IV	51	36	Median	qRT-PCR	OS	7
Yang et al. [17]	China	BRCA	837	I–IV	419	418	Median	qRT-PCR	OS	7
Yang et al. [19]	China	CRC	166	NA	83	83	Median	qRT-PCR	OS	5
Zhang et al. [20]	China	LC	101	I–III	51	50	Median	qRT-PCR	OS	7

*NA* not available, *DFS* disease-free survival, *OS* overall survival, *PDAC* pancreatic ductal adenocarcinoma, *GC* gastric cancer, *KIRP* Kidney renal papillary cell carcinoma, *OC* ovarian cancer, *CRC* colorectal carcinoma, *BLCA* bladder cancer, *HCC* hepatocellular carcinoma, *BRCA* breast cancer, *LC* lung cancer

### FAM83H-AS1 expression significantly correlated with OS

**Fig. 2:**
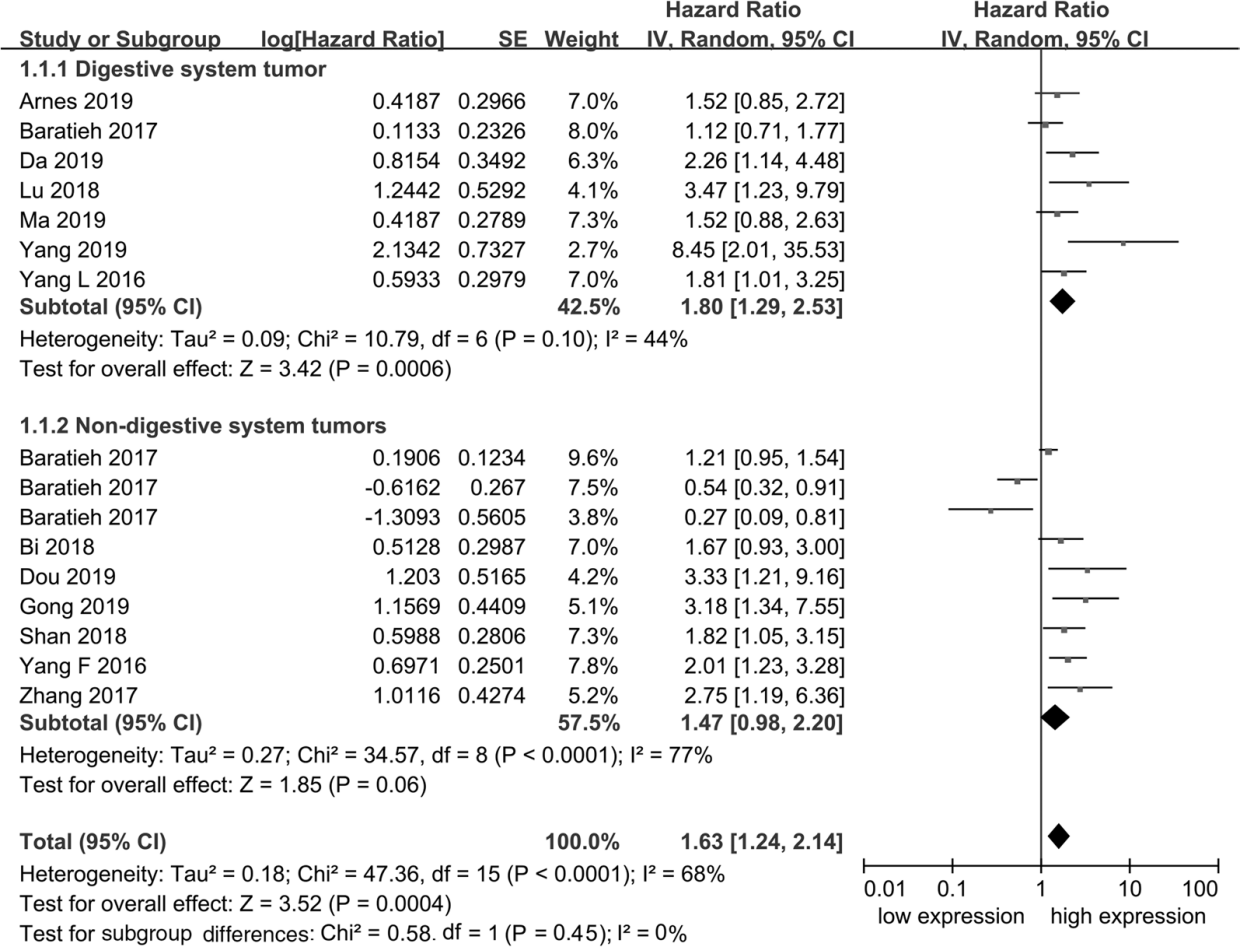
Forest plots of the included literatures evaluating the association between FAM83H-AS1 expression with overall survival (OS)

13 of 14 studies including 2650 patients assessed the HR and 95% CI of OS. Statistical results of all studies are shown in a forest plot in Fig. 2. The random effects model was selected to estimate the pooled HR because no significant heterogeneous among the studies was found (*I*^2^= 68%, *P *< 0.001). The merged results indicated that high expression of FAM83H-AS1 was significantly associated with poor prognosis (pooled HR = 1.63, 95% CI 1.24–2.14, *P *= 0.0004). Patients with high expression of FAM83H-AS1 had a worse OS than those with low expression of FAM83H-AS1.

To analyze the pooled HR among different types of cancer, two subgroups (digestive system tumors and non-digestive system tumors) were established. High FAM83H-AS1 expression was related to poor OS in one of the subgroups (digestive system tumors: pooled HR = 1.65 95% CI 1.30–2.10, *I*^2^= 44%, *P *< 0.0001; non-digestive system tumors: pooled HR = 1.47 95% CI 0.98–2.20, *I*^2^= 77%, *P *= 0.06) (Fig. 2). These results indicate that high expression of FAM83H-AS1 might be a significant prognostic factor of OS and more suitable for application in patients with digestive tumors compared with those with non-digestive tumors.

### Association between FAM83H-AS1 and clinicopathologic characteristics

**Fig. 3:**
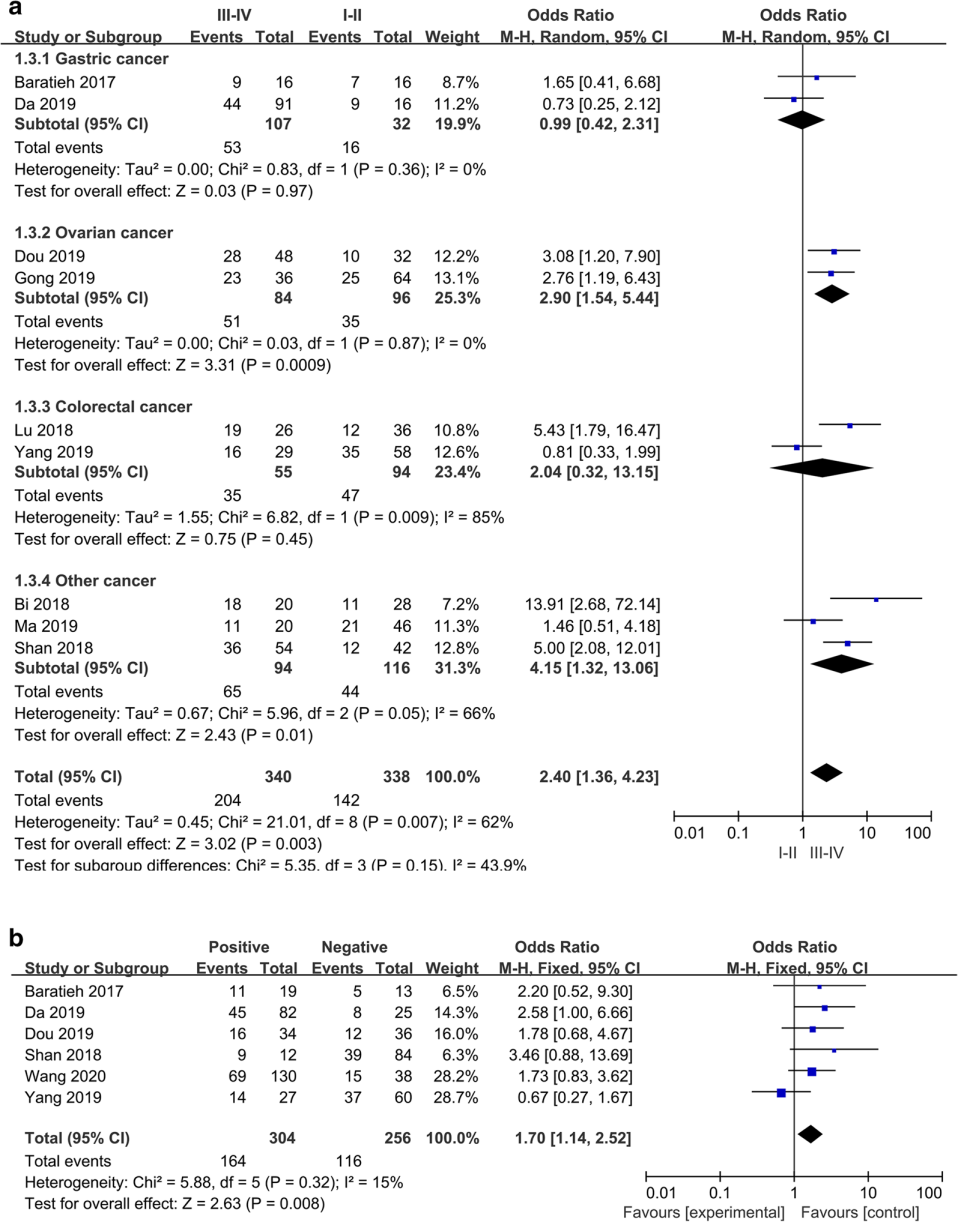
Forest plots of the included literatures evaluating the correlation between FAM83H-AS1 expression and clinicopathological characteristics. **a** TNM stage. **b** Lymph node metastasis

**Fig. 4:**
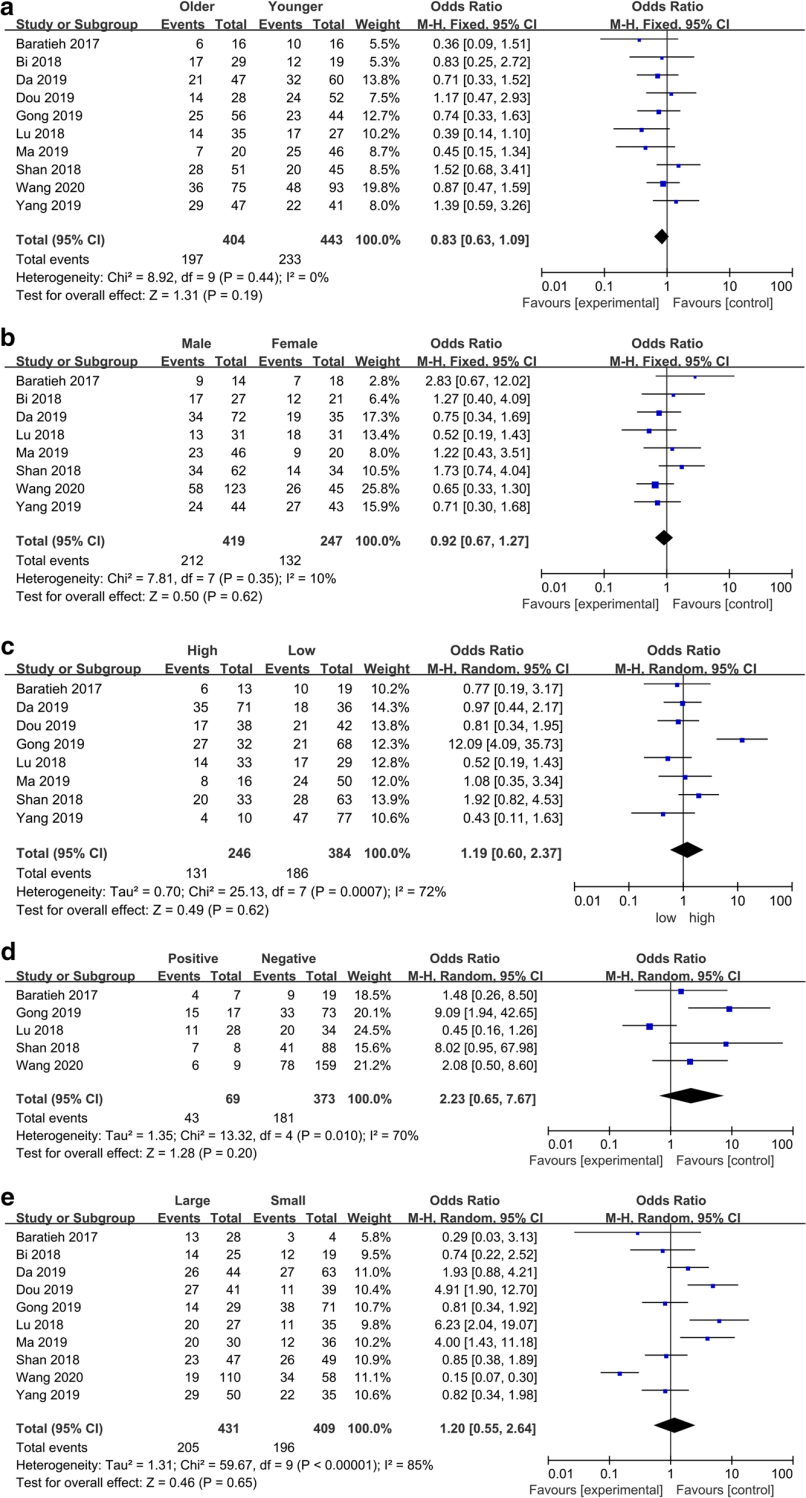
Forest plots of the included literatures evaluating the correlation between FAM83H-AS1 expression and clinicopathological characteristics. **a** Age. **b** Gender. **c** Differentiation. **d** Distant metastasis. **e** Tumor size

Only ten of the eligible studies that contained detailed clinicopathologic characteristics evaluated the correlation between FAM83H-AS1 expression and TNM stage. The results indicated that elevated expression of FAM83H-AS1 was associated with advanced TNM stage (III/IV vs. I/II, OR = 2.40, 95% CI 1.36–4.23, *P *= 0.003) (Fig. 3a). Moreover, six studies examined characteristics of lymph node metastasis. The results demonstrated that elevated FAM83H-AS1 expression was significantly associated with lymph node metastasis (positive vs. negative, OR = 1.70, 95% CI 1.14–2.52, *P *= 0.008) (Fig. 3b). The relationships among elevated expression of FAM83H-AS1 and age, gender, tumor size, differentiation and distant metastasis were also investigated. However, no significant correlation was found between FAM83H-AS1 expression and these characteristics (Fig. 4a–e). Detailed information was shown in Table 2.

**Table 2:** Summary of correlation between FAM83H-AS1 expression and clinicopathological characteristics of cancers

Clinicopathological parameters	Studies	Patients	OR (95% CI)	*P*-value	Heterogeneity		
					*I*^2^ (%)	*P*-value	Model
Age (older vs. younger)	10	847	0.83 (0.63, 1.09)	0.19	0	0.44	Fixed
Gender (male vs. female)	8	666	0.92 (0.67, 1.27)	0.62	10	0.35	Fixed
Tumor size (larger vs. smaller)	10	840	1.20 (0.55, 2.64)	0.65	85	0.0001	Random
Differentiation (poor vs. well)	8	630	1.19 (0.60, 2.37)	0.62	72	0.0007	Random
TNM stage (III + IV vs. I + II)	9	678	2.40 (1.36, 4.23)	*0.003*	62	0.007	Random
LNM (positive vs. negative)	6	560	1.70 (1.14, 2.52)	*0.008*	15	0.32	Fixed
DM (positive vs. negative)	5	442	2.23 (0.65, 7.67)	0.20	70	0.01	Random

Italic values indicate significance of *P* value (*P* < 0.05), which means FAM83H have a significant difference in this clinical index

*LNM* lymph node metastasis, *DM* distant metastasis

### Publication bias and sensitivity analysis

**Fig. 5:**
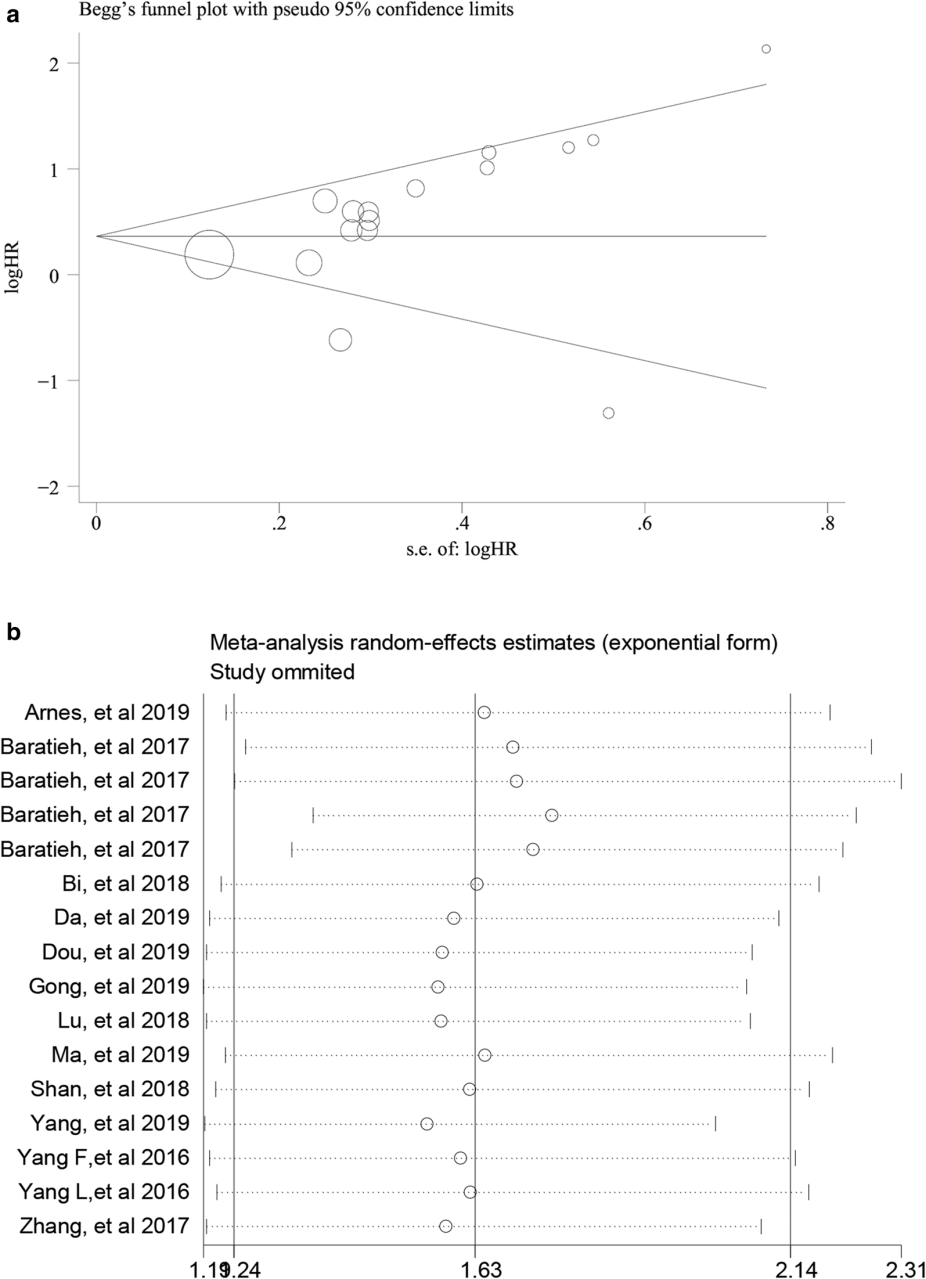
Publication bias and Sensitivity analysis for OS in this meta-analysis. **a** Begg’s funnel plots. **b** Sensitivity analysis

Begg’s test was performed to evaluate the publication bias of the meta-analysis. The results indicated that there was no significant publication bias in this meta-analysis for OS (*P *= 0.093) (Fig. 5a). Additionally, sensitivity analysis revealed no significant change in the pooled HR by eliminating any single study, indicating that the results were stable (Fig. 5b).

## Discussion

LncRNAs, which are encoded by an unstudied region of the human genome, may contain cancer-missing drivers and have attracted attention in recent years as potentially important regulators of carcinogenesis and cancer development. Increasing evidence has indicated that lncRNAs play an important role in various human diseases, especially in malignant tumors [21–24]. Antisense long non-coding RNAs, such as TTN-AS1, IDH1-AS1 and AFAP-AS1, have been reported in tumors and closely related to the prognosis, development, invasion and metastasis of tumors [25–27].

Accumulated evidences exerted that FAM83H-AS1 acted critical roles in various biological and pathological processes. For example, FAM83H-AS1 could ameliorate SpA-mediated inhibition on the osteogenic differentiation of human bone mesenchymal stem cells during osteomyelitis and promote nucleus pulposus cell growth in intervertebral disc degeneration [28, 29]. However, many studies recently showed that FAM83H-AS1 was overexpressed in some tumors and exhibited an oncogenic role in cell proliferation and metastasis [6, 10, 16]. Dou’s study showed that elevated FAM83H-AS1 expression was correlated with radioresistance and poor OS and was used to effectively predict lymph node metastasis in ovarian cancer [16]. Previous studies confirmed that the expression of HuR was positively correlated with the expression of FAM83H-AS1. Furthermore, HuR promoted cell proliferation and metastasis by interacting with lncRNAs in multiple cancers [30, 31]. The underline mechanism was that FAM83H-AS1 could interact with HuR by increasing the stability of the HuR mRNA. Yang’s study revealed that FAM83H-AS1 acted as a prognostic lncRNA in colon cancer and its expression level was significantly elevated in colon cancer tissues. Additionally, FAM83H-AS1 negatively regulated SMAD1/5/9 [32]. One study suggested that FAM83H-AS1 might inhibit TGF-β signaling pathway by downregulating SMAD1/5/9, preventing the anti-tumor effect of TGF-β signaling [32]. Furthermore, numerous studies showed that FAM83H-AS1 played an important role in gastric cancer progression [7]. Overexpressed FAM83H-AS1 was an independent prognostic predictor of OS in gastric cancer; FAM83H-AS1 expression was also significantly related to lymph node metastasis and showed an important value in the differentiation between cancerous and non-cancerous tissues. Increased FAM83H-AS1 expression has recently been reported to predict poor prognosis and promote malignant phenotypes of bladder cancer [10] and pancreatic ductal adenocarcinoma [13]. A research group found that FAM83H-AS1 was a crucial lncRNA expressed at preliminary stage of breast cancer by RNA sequencing in early-stage tumors, suggesting detection of FAM83H-AS1 expression levels in plasma could be a potential diagnostic and prognostic biomarker for early-stage of breast cancer [33]. However, in contrast to other reports, Baratieh’s study showed that upregulated expression of FAM83H-AS1 in kidney renal papillary cell carcinoma led to longer survival rates [14]. Chemotherapy is one of the most effective methods to treat tumors and is the main treatment for some tumors that have the tendency of metastasis and those that have already metastasized. Whereas, some patients tend to develop resistance to chemotherapy drugs. Interestingly, the latest research showed that silence of FAM83H-AS1 sensitized gastric cancer cells to cisplatin and 5-fluorouracil, which were the first-line treatment scheme for gastric cancer [15]. This suggested that designing drugs to reduce the high expression of FAM83H-AS1 might eliminate chemotherapy resistance in some patients with gastric cancer.

The current study presented the first meta-analysis to comprehensively evaluate the relationship between FAM83H-AS1 expression and prognosis and clinicopathological characteristics of tumors. A total of 14 eligible studies containing 2818 patients were enrolled in this meta-analysis. The pooled results revealed that increased FAM83H-AS1 expression was significantly associated with poor prognosis. Furthermore, the high expression of FAM83H-AS1 might be an important prognostic factor for OS in patients with digestive tumors. The subsequent pooled results also demonstrated that high expression of FAM83H-AS1 was associated with lymph node metastasis and high TNM grade of tumors.

Whereas, this study had some limitations. First, few studies on FAM83H-AS1 were available and thus the number of eligible studies that could be included was limited. Furthermore, most of the studies were from China, which might lead to deviation and might represent the clinical characteristics of Chinese patients with tumors. In addition, because of the relatively small sample size, we were unable to aggregate results based on a single type of tumor. Finally, some studies did not directly give the results and we used an indirect method to obtain HRs and 95% CI. Hence, the clinical significance of high FAM83H-AS1 expression might be overestimated. However, the statistical results could be improved with an increased number of follow-up studies.

In summary, FAM83H-AS1 plays a crucial function as an effective predictive biomarker for tumor prognosis. More relevant studies and in-depth data analysis are needed to further confirm the overall diagnostic value of FAM83H-AS1 in cancers.

## Conclusions

Our study first systematically reviewed and estimated the relationship between abnormal FAM83H-AS1 expression and survival and clinical outcomes in patients with tumors. The present results suggested that high expression level of FAM83H-AS1 was associated with poor OS and lncRNA FAM83H-AS1 might be used as a prognostic marker for patients with cancer. Considering the limitations of this study, it is necessary to conduct more large-scale and high-quality studies on various ethnic populations to obtain more value of FAM83H-AS1 in tumors.

## Data Availability

All data are included in this article.

## Abbreviations

LncRNA: Long non-coding RNA
FAM83H-AS1: Family with sequence similarity 83 member H antisense RNA 1
NA: Not available
DFS: Disease-free survival
OS: Overall survival
PDAC: Pancreatic ductal adenocarcinoma
GC: Gastric cancer
KIRP: Kidney renal papillary cell carcinoma
OC: Ovarian cancer
CRC: Colorectal carcinoma
BLCA: Bladder cancer
HCC: Hepatocellular carcinoma
BRCA: Breast cancer
LC: Lung cancer
LNM: Lymph node metastasis
DM: Distant metastasis
